# A reexamination of theoretical arguments that indirect selection on mate preference is likely to be weaker than direct selection

**DOI:** 10.1002/evl3.276

**Published:** 2022-02-12

**Authors:** James D. Fry

**Affiliations:** ^1^ Department of Biology University of Rochester Rochester New York 14627

**Keywords:** Evolutionary theory, genetic correlation, linkage disequilibrium, models, natural selection, quantitative genetics, sexual selection

## Abstract

Female preference for male ornaments or displays can evolve by indirect selection resulting from genetic benefits of mate choices, or by direct selection resulting from nongenetic benefits or selection on sensory systems occurring in other contexts. In an influential paper, Kirkpatrick and Barton used a good‐genes model and evolutionary rates estimated from the fossil record to conclude that indirect selection on preference is likely to be weak compared to typical strengths of direct selection. More recent authors have extrapolated from Kirkpatrick and Barton's conclusions to suggest that the presence of preference‐trait genetic correlations in equations for indirect but not direct selection gives a purely theoretical basis to the conclusion that the former is likely to be weaker than the latter. Here, I challenge these views, and argue that the relative importance of direct and indirect selection on preference is an empirical issue that defies simple generalizations. First, I show that Kirkpatrick and Barton based their conclusion on a questionable claim about typical rates of evolution due to direct selection. Second, I argue that claiming that direct selection on preference is stronger than indirect selection because only equations for the latter contain a genetic correlation mistakes the mathematical simplicity with which direct selection is usually represented for evidence regarding its magnitude. By comparing a simple equation for the selection response of preference caused by somatic (“direct”) benefits to Kirkpatrick and Barton's result for the response to indirect selection, I show that indirect selection on preference is not inherently weaker than direct selection. I also point out an important but overlooked reason why selection on preference under the sensory bias hypothesis can be expected to be less effective in the long run than that from either somatic or genetic benefits of mate choices.

**Impact Summary**

Females of many animal species scrutinize potential mates based on their songs, colors, or displays. Why such preferences have evolved has puzzled biologists since Darwin. One long‐standing hypothesis is that courtship traits give information about a male's genetic quality, so that males with superior versions of the traits are likely to father relatively healthy and vigorous offspring. An alternative possibility is that courtship traits signal a male's ability to provide nongenetic benefits to the female or her offspring, such as high fertility or parental care. An additional, nonadaptive hypothesis is that mate preferences are merely by‐products of features of female nervous systems that evolved for other reasons. The debate over these hypotheses has been strongly influenced by claims that on theoretical grounds, the genetic quality hypothesis is inherently less feasible than the other two. Here, I show that these arguments are flawed, and that of the three hypotheses, it is the by‐product hypothesis that suffers from a theoretical weakness.

There are several hypotheses for why females in many animal species prefer to mate with males with exaggerated courtship traits (reviewed in Kirkpatrick and Ryan 1991; Maynard Smith 1991; Andersson 1994; Kokko et al. 2003; Jones and Ratterman 2009; Rosenthal 2017). Characteristics of a male's ornaments, displays, or vocalizations might be indicators of nongenetic benefits he can provide, such as high fertility or parental care (although such benefits are often called “direct,” this term might be taken to imply that the benefits go to the female rather than to her offspring; to avoid possible confusion, I will refer to all nongenetic benefits as “somatic”; cf. Jia and Greenfield 1997). Alternatively, male traits preferred by females could be indicators of potential genetic benefits to the offspring (often called “indirect benefits”). According to the good genes hypothesis (Williams 1966; Kodric‐Brown and Brown 1984; Maynard Smith 1991), males with preferred traits father offspring with relatively high viability and vigor—for example, because they have relatively few deleterious mutations (Kirkpatrick 1996; Houle and Kondrashov 2002), or have genes conferring resistance to common parasites (Hamilton and Zuk 1982). According to the Fisherian model (Fisher 1958), in contrast, the inheritance of high mating success by the sons of choosy females may be sufficient to favor further amplification of preference, or at least maintain preference at its current level (Lande 1981; Pomiankowski et al. 1991). An alternative, nonadaptive hypothesis, variously called the sensory bias, perceptual bias, or preexisting bias hypothesis, is that mate preference evolves as a by‐product of selection on female sensory systems that occurs in other contexts, such as foraging or predator avoidance (Ryan 1990; Kirkpatrick and Ryan 1991; Ryan and Cummings 2013).

Although the debate about why females prefer to mate with males with particular traits must ultimately be settled with empirical data, theoretical arguments have figured prominently in discussions of hypotheses of mate choice (e.g., Lande 1981; Kirkpatrick 1982, 1985; Grafen 1990; Iwasa et al. 1991; Pomiankowski et al. 1991; Rowe and Houle 1996; Kirkpatrick and Barton 1997). One such argument is that indirect selection on mate preference, that resulting from genetic benefits, is likely to be weaker than direct selection, that resulting from somatic benefits and, according to some authors (Kirkpatrick and Ryan 1991; Kirkpatrick and Barton 1997), sensory bias. This conclusion was reached in an influential theoretical paper by Kirkpatrick and Barton (1997), and was later arrived at by a somewhat different line of reasoning by Cameron et al. (2003). It has since become widely accepted (e.g., Kokko et al. 2006; Hettyey et al. 2010; Kuijper et al. 2012; Ryan and Cummings 2013; Kiyose et al. 2015; Rosenthal 2017; Fitzpatrick and Servedio 2018; Suzaki et al. 2018; Svensson 2019; Kelly and Adam‐Granger 2020; Madjidian et al. 2020). For example, in a review of sexual selection theory, Kuijper et al. (2012) cite both Kirkpatrick and Barton (1997) and Cameron et al. (2003) in support of the statement that “even slight costs can override indirect benefits of choosiness, leading to the theoretical expectation that sexual selection driven only by indirect benefits of choosiness is rare in nature.” Similarly, Fitzpatrick and Servedio (2018), citing Kirkpatrick and Barton (1997), write that “it is well known that indirect selection tends to be weaker than direct selection because it is mediated by the strength of imperfect genetic correlations” (as will be shown later, this more accurately summarizes the argument of Cameron et al. than that of Kirkpatrick and Barton).

Here, I challenge these views, and argue that the relative importance of direct and indirect selection on preference is an empirical issue that defies theoretical generalization. First, I show that Kirkpatrick and Barton (1997) based their conclusion on a questionable claim about typical strengths of direct selection; their estimated rate of preference evolution under a good‐genes model is not lower than typical evolutionary rates. Second, I argue that claiming that direct selection on preference is stronger than indirect selection because only equations for the latter contain a genetic correlation (Cameron et al. 2003; Fitzpatrick and Servedio 2018; cf. Mead and Arnold 2004; Kokko et al. 2006) mistakes the mathematical simplicity with which direct selection is usually represented for evidence regarding its magnitude. By comparing a simple equation for the selection response of preference caused by somatic benefits to Kirkpatrick and Barton's (1997) result for the response to indirect selection, I show that indirect selection on preference is not inherently weaker than direct selection. I also point out an important but overlooked reason why selection on preference under the sensory bias hypothesis can be expected to be less effective in the long run than that from either somatic or genetic benefits of mate choices.

## The Argument of Kirkpatrick and Barton (1997)

Kirkpatrick and Barton (1997) used a quantitative‐genetic approach to derive an expression for the strength of selection on preference under a good genes model. They assumed that females vary in preference (*P*), and males vary in an indicator trait (*T*), such that a female's value of *P* is correlated with her mate's value of *T*. Under these assumptions, if both *P* and *T* are heritable, and *T* is genetically correlated with overall fitness, mate choice will cause a genetic correlation to arise between *P* and *T*, which in turn causes *P* to become genetically correlated with fitness. The response to selection of any trait is given by its genetic covariance with relative fitness *W* (Robertson 1966; Queller 2017; Walsh and Lynch 2018), which for preference is given by

(1) $$\Delta_{I}=r_{PW}h_{P}\sqrt{G_{W}}.$$

Here, *r_PW_* is the genetic correlation between preference and fitness, *h_P_* is the square‐root of the heritability of preference (which was assumed to have unit phenotypic variance), and *G_W_* is the additive genetic variance in relative fitness (i.e., fitness scaled to a mean of one); the subscript *I* indicates that we are considering the response of preference to indirect selection only.

Because indirect selection is based on the genetic correlation resulting from associations between preference loci and trait loci, for the purpose of calculating *Δ_I_* we can disregard any other causal route between preference loci and fitness, giving *r_PW_* = *r_PT_* ·*r_TW_*. Furthermore, as shown by Kirkpatrick and Barton (1997; see also Pomiankowski and Iwasa 1993), the genetic correlation between *P* and *T* can be well approximated by

(2) $$r_{PT}\approx 1\;/\;{}_{2}\rho_{PT\left(\mathrm{mates}\right)}h_{P}h_{T}.$$

Here, *ρ_PT_*_(mates)_ is the correlation between a female's value of *P* and her mate's value of *T*, a measure of the effectiveness of mate choice. Substituting these results into (1) gives Kirkpatrick and Barton's equation (9):

(3) $$\Delta_{I}=1\;/\;{}_{2}\rho_{PT\left(\text{mates}\right)}r_{TW}h_{T}\sqrt{G_{W}}{h_{P}}^{2}.$$

Using parameter estimates from the literature, and setting both *ρ_PT_*_(mates)_ and *r_TW_* to 1, the authors concluded that *Δ_I_* is unlikely to exceed 0.035, that is, a selection response of preference of 3.5% of a phenotypic standard deviation per generation.

Kirkpatrick and Barton's derivation of equations (2) and (3) was an important contribution, and even though one could quibble with the estimate of *G_W_* they used (cf. Houle and Kondrashov 2002), overall their parameter choices were generous to the good‐genes hypothesis. The problem was in the method they used to estimate the strength of direct selection on preference, *Δ_D_*. Citing Gingerich (1983) and Stearns (1992) (their references 30 and 31), they wrote:

Estimates of *Δ_D_* are not available for any preference. Direct selection on other kinds of characters, however, can cause values of more than an order of magnitude larger than 0.035. [This follows from observed rates of evolution (30,31) and the relation *Δ_D_* = 10^–4^ *d*/CV_P_, where *d* is the character's evolutionary rate measured in darwins (30), and CV_P_ is its phenotypic coefficient of variation expressed as a percentage, and assuming one generation per year on average]. Thus, direct selection on preference genes may overwhelm indirect selection. On the other hand, even weak indirect selection will be important if preference genes are virtually selectively neutral, that is, free of direct selection.

Here, the authors seem to be arguing that rates of character evolution are so high that a source of selection that changes the mean of a trait by an average of 0.035 standard deviations per generation is likely to be relatively insignificant. To attempt to verify this conclusion, I used the mean rates of evolution in darwins reported in Gingerich (1983) to calculate per‐generation changes following the authors’ method, conservatively assuming CV_P_ = 2.5% (the values from Stearns mostly repeat those from Gingerich). The highest value I obtained, that for recent (≤300 years BP) colonization events in mammals, was 0.015 standard deviations per generation; for post‐Pleistocene mammals the value was 0.00015, and for the older vertebrate and invertebrate fossil records <0.00001 (note that all of these estimates, like the 0.035 figure, imply nothing about the direction of evolution, because darwins are reported as absolute values). It is possible that Kirkpatrick and Barton (1997) inappropriately used data from artificial selection experiments, or used the maximum values for each category rather than the means (even in this case, only historical colonization events would have yielded the high rates they claim). Whatever the explanation, selection causing evolution of 0.035 standard deviations per generation over a sustained period appears to be rare. The most we can conclude is that direct selection on morphological traits is *sometimes* stronger than a plausible upper limit for indirect selection on preference.

Of course, it is unrealistic to imagine that either *ρ_PT_*_(mates)_ or *r_TW_* are close to 1. But even if *Δ_I_* were two orders of magnitude lower than Kirkpatrick and Barton's (1997) estimated upper limit, it would still be higher than the means from each of Gingerich's (1983) fossil datasets (including that from post‐Pleistocene mammals, which appears to be the dataset least subject to the biases that he discusses). Consequently, even if we were to assume that the strength of selection on morphological traits is a good proxy for that of direct selection on preference, Kirkpatrick and Barton's (1997) comparison does not give evidence that indirect selection on preference is typically weaker than direct selection. As a result, the authors’ implication that preference genes would have to be under untypically weak direct selection in order for indirect selection to be important is not justified.

## The Genetic Correlation Argument

Interestingly, authors summarizing Kirkpatrick and Barton's (1997) reasoning have sometimes overlooked those authors’ use of estimates of evolutionary rates, instead substituting a simpler argument that Kirkpatrick and Barton did not make: that the involvement of a genetic correlation in indirect selection but not direct selection should make the former weaker on average (Cameron et al. 2003; Fitzpatrick and Servedio 2018). A slightly different, but essentially equivalent, interpretation of Kirkpatrick and Barton (1997) is that because equation (3) summarizes “a long causal pathway, from preference to ornament to total fitness . . . a weak link anywhere in the pathway means that the whole pathway will also be weak” (Mead and Arnold 2004; see also Kokko et al. 2006).

The first authors making an argument like these appear to have been Cameron et al. (2003), who represented responses of preference to direct and indirect selection as follows:

(4A) $$\Delta_{D}=\beta_{P}G_{P},$$

(4B) $$\Delta_{I}=\beta_{T}G_{PT}.$$

Here, *G_P_* is the genetic variance of preference, *G_PT_* is the genetic covariance between preference and the male trait, and *β_P_* and *β_T_* are the selection gradients on preference and the trait, respectively. The authors argued that because “under most conditions the additive genetic variance in female preference is expected to be greater than the additive genetic covariance between it and the male trait,” we should expect *Δ_D_* to usually be greater than *Δ_I_*. (The reason that genetic covariances are expected to be on average lower than genetic variances can be understood by decomposing the genetic covariance into its component parts: *G_PT_* = *r_PT_* (*G_P_* ·*G_T_*)^1/2^. Because we would expect *r_PT_* to be substantially less than 1, if we add the assumption that *G_P_* ≈ *G_T_*, it follows that *G_PT_* < *G_P_*. If we also make the assumption that *β_P_* ≈ *β_T_*, we reach the authors’ conclusion.)

One problem with Cameron et al.’s argument is that selection gradients and genetic variances vary considerably among traits, so need not be approximately equal for any particular pair of traits. For the argument to be strictly valid, we would have to view *β_P_* and *β_T_* as random samples from a common distribution of selection gradients, and *G_P_* and *G_T_* as random samples from a common distribution of genetic variances. If we were then to add the assumption that this “sampling” is done independently for every species, then it would have to be true that ${\bar{\beta }}_{P}={\bar{\beta }}_{T}$ and ${\bar{G}}_{P}={\bar{G}}_{T}$. But *P* and *T* represent real traits, and very different ones; we are not entitled to assume that their selection gradients and genetic variances are equal, either on average or in any particular species.

But there is an additional, and more important, problem with Cameron et al.’s (2003) argument, as well as those of Mead and Arnold (2004) and Fitzpatrick and Servedio (2018). In representing the response of preference to selection by equations like (3) and (4B), we are making use of the fact that mate choice can cause genes that affect preference to become associated with fitness through a particular *mechanism*, the creation of interlocus associations. In contrast, in representing the response of preference to selection by an equation like (4A), we are simply ignoring the mechanism by which preference genes become associated with fitness. Any chain of causation by which a gene or trait influences fitness, however, could be mathematically decomposed into several steps (in fact an arbitrary number of such steps), each of which could be represented by a correlation or some other parameter. The fact that we choose not to do so for the various hypothesized sources of direct selection on preference (e.g., somatic benefits) does not establish anything about the strength of the resulting selection.

To illustrate this point, suppose that we modify Kirkpatrick and Barton's (1997) model to let the male trait also be correlated with the somatic fitness effect *S* that a male has on its mate or its offspring. *S* has phenotypic variance *V_S_*, and the phenotypic correlation between *T* and *S* is *ρ_TS_*. Under these assumptions, the regression of her mate's *S* on a female's value of *P* will be *ρ_PT_*_(mates)_ *ρ_TS_* $\sqrt{V_{S}}$. Regardless of whether the fitness benefits go to the female or her offspring, the resulting response of preference to selection is

(5) $$\Delta_{D\left(SB\right)}=1\;/\;{}_{2}\rho_{PT\left(\mathrm{mates}\right)}\rho_{TS}\sqrt{V_{S}}\;{h_{P}}^{2}.$$

(If the benefits go to the female, the ½ arises because selection on preference does not occur in males; if they go to the offspring, the ½ is the degree of relatedness between mothers and offspring.) Note that (5) is very similar to Kirkpatrick and Barton's (1997) result for indirect selection (my eq. 3), with *ρ_TS_* replacing *r_TW_* ·*h_T_*, and *V_S_* replacing *G_W_*.

To compare the strengths of indirect and direct selection on preference, we can take the ratio of *Δ_I_* to *Δ_D_*_(_ *_SB_*_)_ (I use absolute values to account for the possibility that direct and indirect selection act in opposite directions):

(6) $$\left|\frac{\Delta_{I}}{\Delta_{D(\mathrm{SB})}}\right|=h_{T}\left|\frac{r_{TW}}{\rho_{TS}}\right|{\left(\frac{G_{W}}{V_{S}}\right)}^{1/2}.$$

On one hand, *h_T_* must be less than 1 (although not so much so as the heritability itself). On the other hand, phenotypic and genetic correlations tend to be similar (Kruuk et al. 2008; Sodini et al. 2018), so with the exception of cases where the male trait has an obvious connection to a somatic benefit he can deliver (e.g., courtship feeding), there is no basis for assuming that |*r_TW_* | will on average be smaller than |*ρ_TS_* |. Moreover, although the genetic variance for fitness is likely to be nontrivial in most species (e.g., Merilä and Sheldon 2000; Blomquist 2010; Kosova et al. 2010; Wolak et al. 2018), we would expect *V_S_* to vary considerably depending on the mating system of the species under consideration. In species in which males defend feeding territories and care for young, *V_S_* might approach or exceed *G_W_*, but in lekking species, in which males interact only briefly with females and offer no obvious somatic benefits to females or their offspring, we might reasonably expect *V_S_* << *G_W_*. Therefore whether (6) is greater or less than 1 is an empirical issue whose answer is likely to depend on the species being considered. (It is also interesting to note that neither the genetic correlation between preference and the male trait nor any other quantitative‐genetic parameter involving preference appears in eq. 6).

The above results are based on comparing the contributions of genetic and somatic benefits to the selection response of preference at a given point in time. One might also ask whether, if preference evolves to an equilibrium at which its benefits are balanced by costs, somatic benefits are inherently more influential in determining the resulting mean preference than genetic benefits. This issue was addressed by Iwasa and Pomiankowski (1999), who modeled evolution of a condition‐dependent ornament whose size is an increasing function of the product of the male's investment in it and his condition or quality, *Q*. Males provide a somatic fitness benefit (e.g., parental care) to females or their offspring proportional to *Q*; because *Q* is positively correlated with viability in both sexes, females that choose high‐quality males also receive genetic benefits, as long as quality is heritable. Females, however, can only assess a male's quality through its correlation with ornament size; moreover, larger ornaments reduce male fitness, disproportionally so for low‐quality males. The authors show that, given these assumptions, female preference and ornament size evolve to a joint equilibrium at which the ornament is exaggerated and females prefer males with above average ornament size. After a simple reparameterization (see Appendix), the equilibrium for preference can be written as

(7) $$\hat{\bar{P}}\;\propto \;\frac{s\;V_{Q}\;+\;\beta_{Q\;}G_{Q}\;}{c}.$$

Here, *V_Q_* and *G_Q_* are the phenotypic and genetic variances of quality, *s* measures the strength of somatic benefits (the increase in female or offspring fitness per unit increase of male quality), *c* measures the cost of choosiness (minimized at *P* = 0, when preference is absent), and *β_Q_* is the selection gradient on quality. From (7), genetic benefits will have a greater influence on the equilibrium preference than somatic benefits whenever *G_Q_*/*V_Q_*, the heritability of quality, exceeds |*s*|/*β_Q_*. As was the case for expression (6), this result does not lend itself to generalizations about the relative importance of direct and indirect selection on preference, because |*s*| would be expected to vary considerably depending on the mating system of the species under consideration. In lekking species, we might reasonably expect that the effect of a male's quality on the fitness of his mate or offspring will be trivial compared to the effect of an individual's quality on its *own* fitness, implying |*s*| << *β_Q_*.

Expression (7) also illustrates that if we wish to distinguish between somatic and genetic benefits as explanations for why females choose males based on a particular trait, knowing the magnitude of search costs will tell us very little. For example, if we found that search costs are small, this would indicate that a high level of choosiness could be maintained by only modest benefits, but would not give information on the nature of the benefits. That some authors seem to regard search costs as especially detrimental to the good genes hypothesis (e.g., Kotiaho and Puurtinen 2007; Kuijper et al. 2012) simply reflects these authors’ apparent assumption that, in effect, the first term of the numerator of (7) is likely to be much larger than the second term. (It is true, however, that the Fisherian runaway is especially sensitive to search costs [see Kuijper et al. 2012].)

Finally, although I have been referring to “benefits” of preference, males often harm females or their offspring (e.g., Linder and Rice 2005; Perry and Rowe 2015). For modeling how preference will evolve, however, it is inconsequential whether interactions with males have net positive or negative effects on female fitness, aside from through the provision of sperm. A positive value of *s* in (7), or of *ρ_TS_* in (5), could arise either because *Q* or *T* are positively associated with the somatic benefits that males provide, or because they are negatively associated with the harm that males cause. Similarly, a negative value of *s* does not require that males harm females; it could simply mean that *Q* or *T* are negatively associated with the somatic benefits that males provide. (From (7), in the case of negative *s*, genetic and somatic benefits would tend to cancel each other, possibly resulting in lower choosiness at equilibrium than if either acted alone.)

## An Overlooked Limitation of the Sensory Bias Hypothesis

According to the sensory bias (or perceptual bias) hypothesis, the evolution of mate preference is strongly influenced by selection occurring in other contexts, such as foraging or predator avoidance (Ryan 1990, 1998; Kirkpatrick and Ryan 1991; Ryan and Cummings 2013). At first glance, this should not be controversial: one expects that males will signal in sensory modalities that females are capable of perceiving, and that the sensory abilities of females of a species will be to a considerable extent the product of past selection in contexts other than mating. Proponents of the sensory bias hypothesis, however, sometimes go further, suggesting that selection in contexts such as predator avoidance may sometimes keep mating behavior from evolving to its optimum based on its own fitness effects, for example, by causing suboptimal mate choices (Ryan and Cummings 2013).

Kirkpatrick and Barton (1997; see also Kirkpatrick and Ryan 1991) included sensory bias among possible sources of direct selection on preference, and thus implied that selection on preference resulting from sensory bias is likely to be more effective than that resulting from genetic benefits (cf. Ryan and Cummings 2013). As some authors have recognized, however, for a selection response of preference to occur under the sensory bias hypothesis there must be a genetic correlation—hypothesized to be caused by pleiotropy rather than by interlocus associations—between preference and the foraging or predator‐avoidance trait (Fuller et al. 2005; Fuller 2009; Alem et al. 2013; Cole and Endler 2018). In other words, selection on preference resulting from sensory bias is more appropriately represented by an equation like (4B), where *T* is now the foraging or predator‐avoidance trait (either a measure of behavior or of some feature of the sensory system), than one like (4A). Somewhat ironically, then, if one adopted the reasoning of Cameron et al. (2003) and Fitzpatrick and Servedio (2018), who regarded themselves as paraphrasing Kirkpatrick and Barton (1997), one would conclude that selection on preference caused by sensory bias should be expected to be relatively weak (neither Cameron et al. nor Fitzpatrick and Servedio mentioned the sensory bias hypothesis, it should be noted).

As argued in the previous section, however, the conclusion that (4A) is likely to be greater than (4B) holds only on average, and is not necessarily true for any particular pair of traits. But the theory of selection on correlated characters (Lande 1979; Via and Lande 1985; Falconer and Mackay 1996) provides a more consequential reason why selection on nonmating behaviors might be expected to have a relatively limited impact on mate preference in the long run compared to selection resulting from the effects of preference itself. In models of evolution of correlated characters, unless genetic variation in some dimension of multivariate space is lacking (such as would occur if the genetic correlation between a pair of traits were exactly ±1), the only possible equilibrium occurs when all traits are at their respective optima (Lande 1979; Via and Lande 1985). In a two‐trait model, for example, it would not be possible for there to be an equilibrium where one trait is at its optimum and the other is not, because without directional selection on the first trait, there would be no correlated response in the second trait to hinder its progress toward its own optimum. Of course, it is possible that foraging or predator‐avoidance behaviors rarely get close to their optima, and so continue to generate maladaptive correlated responses of preference over long periods (by the same token, selection resulting from mate choices would generate maladaptive correlated responses of the foraging or predator avoidance trait). My main point is that, to establish that selection on foraging or predator‐avoidance behavior is currently opposing optimization of mate choices in a population, one would need to demonstrate not only that the two types of behavior are genetically correlated, as has been recognized, but that directional selection on the former is ongoing, which does not appear to have been widely recognized.

## Conclusion

The claim is often made that indirect selection on preference is expected to be weaker than direct selection. Although this is undoubtedly true for particular species (arguably including most of those with biparental care), here I have shown that there is no general theoretical reason why it should be so. Neither the argument of Kirkpatrick and Barton (1997) based on the fossil record nor that of Cameron et al. (2003) and other authors based on genetic correlations withstands careful scrutiny (and the former is not a purely theoretical argument). Moreover, my expressions (6) and (7) make explicit why, in species in which males have limited opportunity to directly influence the fitness of the female or her offspring, genetic benefits of mate choices are likely to be at least as important as somatic benefits, claims about the inherent weakness of indirect selection notwithstanding. I have also pointed out that in order to explain the long‐term maintenance of maladaptive mating preferences, the sensory bias hypothesis would require not only the existence of a genetic correlation between preference and a second trait, but some factor that prevents the second trait from reaching its optimum. As a result, if any hypothesis of mate choice can be said to have an a priori theoretical weakness, it is the sensory bias hypothesis.

Although many workers continue to test predictions of the good‐genes and related hypotheses (e.g., Sardell et al. 2014; Montoya and Torres 2015; Howie et al. 2019), the claim that indirect selection on preference is expected to be relatively weak in some general or theoretical sense continues to be made (e.g., Kuijper et al. 2012; Ryan and Cummings 2013; Rosenthal 2017; Fitzpatrick and Servedio 2018; Svensson 2019; Kelly and Adam‐Granger 2020). The relative importance of direct and indirect selection on preference is an empirical question that can only be answered by careful comparisons of somatic and genetic benefits of mate choices in species representing a wide range of mating systems.

## DATA ARCHIVING

Data sharing not applicable to this article as no datasets were generated or analyzed during the current study.

## Appendix

My expression (7) is based on equation (14) in Iwasa and Pomiankowski (1999). For simplicity, I have left out a few parameters that act as scale factors (e.g., the strength of selection on ornament size). I have also ignored the parameter γ; if γ > 2, search costs increase exponentially with increasing preference, making a stable equilibrium with nonzero preference possible. The strict proportionality implied by (7) holds if γ = 3; otherwise, the equilibrium preference is proportional to the term on the right raised to the power of 1/(γ – 2) (see the first equation on p. 101 of Iwasa and Pomiankwoski [1999], and eq. 10A in Iwasa and Pomiankowski [1994]).

The most significant change I have made, however, is to approximate *w*, the parameter representing “mutational bias” for quality, by *β_Q_ G_Q_*. This is reasonable because *w* is the amount by which mutation reduces mean quality per generation; at equilibrium, it must be exactly counterbalanced by the selection response of quality, which at equilibrium will be primarily direct. Some evolution of quality could also occur as a correlated response to selection on the other three traits in the model; these are female preference *P*, and the male traits *T* and *T*ʹ, with ornament size being given by the relationship *S* = *T* *+* *T*ʹ*Q* (Iwasa and Pomiankowski 1994, 1999). At equilibrium, however, these traits will be close to their direct selection optima, so the overall correlated response of *Q* to selection on the other traits would be expected to be small.

One way to understand the necessity for the mutational bias parameter is that, if we assume that quality is closely related to fitness and therefore always under positive selection, no equilibrium would be possible without some force opposing its selection response. The authors point out (Iwasa et al. 1991; Iwasa and Pomiankowski 1999), however, that even though they call *w* the mutational bias parameter, it could equally well represent the reduction in mean quality caused by environmental change—for example, that caused by evolution of a population's natural enemies, or random movement of trait optima.
